# Realizing privacy preserving genome-wide association studies

**DOI:** 10.1093/bioinformatics/btw009

**Published:** 2016-01-14

**Authors:** Sean Simmons, Bonnie Berger

**Affiliations:** Department of Mathematics and CSAIL, MIT, Cambridge, MA, USA

## Abstract

**Motivation:** As genomics moves into the clinic, there has been much interest in using this medical data for research. At the same time the use of such data raises many privacy concerns. These circumstances have led to the development of various methods to perform genome-wide association studies (GWAS) on patient records while ensuring privacy. In particular, there has been growing interest in applying differentially private techniques to this challenge. Unfortunately, up until now all methods for finding high scoring SNPs in a differentially private manner have had major drawbacks in terms of either accuracy or computational efficiency.

**Results:** Here we overcome these limitations with a substantially modified version of the neighbor distance method for performing differentially private GWAS, and thus are able to produce a more viable mechanism. Specifically, we use input perturbation and an adaptive boundary method to overcome accuracy issues. We also design and implement a convex analysis based algorithm to calculate the neighbor distance for each SNP in constant time, overcoming the major computational bottleneck in the neighbor distance method. It is our hope that methods such as ours will pave the way for more widespread use of patient data in biomedical research.

**Availability and implementation:** A python implementation is available at http://groups.csail.mit.edu/cb/DiffPriv/.

**Contact:** bab@csail.mit.edu

**Supplementary information:** Supplementary data are available at *Bioinformatics* online.

## 1 Introduction

Genome-wide association studies (GWAS) are a cornerstone of genotype–phenotype association in humans. These studies use various statistical tests to measure which polymorphisms in the genome are important for a given phenotype and which are not. With the increasing collection of genomic data in the clinic, there has been a push towards using this information to validate classical GWAS findings and generate new ones (Weber *et al.*, 2009). Unfortunately, there is growing concern that the results of these studies might lead to loss of privacy for those who participate in them (Erlich and Narayanan, 2014; Homer *et al.*, 2008; Lumley and Rice, 2010).

These privacy concerns have led some to suggest using statistical tests that are differentially private (Jiang *et al.*, 2014; Johnson and Shmatikov, 2013; Tramer *et al.*, 2015; Uhler *et al.*, 2013; Wang *et al.*, 2014; Yu and Ji, 2014; Yu *et al.*, 2014). On the bright side, such methods, properly used, can help ensure a high degree of privacy. Moreover, recent work has suggested that differentially private methods can be used to help avoid overfitting and related problems that plague much of biomedical science (Dwork *et al.*, 2015). These gains, however, have traditionally come at a high cost in utility and efficiency. Moreover, since the genome is extremely high dimensional, this cost is especially pronounced, as was noted in previous works (Uhler *et al.*, 2013). In order to help balance utility and privacy, new methods are needed that provide greater utility than current methods while achieving equal or greater privacy.

Here we improve upon the state of the art in differentially private GWAS. We build on previous work (Johnson and Shmatikov, 2013), which applied the ideas of differential privacy to common analysis approaches in case-control GWAS. In particular, we show how to use non-convex optimization to overcome many of the limitations of their method for picking high scoring SNPs in a differentially private way, making the approach computationally tractable (Johnson and Shmatikov 2013; Yu *et al.*, 2014). Unlike previous work (Yu and Ji, 2014), we are able to achieve this while protecting the genomic data of all study participants. Second, we demonstrate how to give improved significance estimates for the chosen SNPs using input, as opposed to output, perturbation-based methods. Taken together, these results substantially advance our ability to perform differentially private GWAS.

### 1.1 Previous work

Previous works have looked at using differentially private versions of the Pearson χ^2^ and allelic test statistics (defined below) to find high scoring SNPs, beginning with the work of Uhler *et al.* Since then numerous others have worked on this problem (Jiang *et al.*, 2014; Johnson and Shmatikov, 2013; Wang *et al.*, 2014; Yu and Ji, 2014; Yu *et al.*, 2014), and there has even been a competition where teams attempted to improve on the state of the art (Jiang *et al.*, 2014). There have also been suggestions of using similar perturbation based techniques in other areas of biomedical data analysis (Wieland *et al.*, 2008).

Previous works focused on using three different approaches for picking high scoring SNPs—namely a neighbor distance based one, a Laplacian mechanism based one, and a score-based one (see Yu *et al.*, 2014 for details). These studies have suggested the score-based method is an improvement on the Laplacian-based method. The relation between the neighbor-based method and the other two is more complicated, however. Though it often outperforms them, it turns out that the ranking of SNPs favored by the neighbor method is not always the same as that favored by the other methods. Moreover, the neighbor method is more computationally demanding, leading others to use approximate versions of it (Yu *et al.*, 2014).

Previous work on speeding up the neighbor method has assumed that the control groups genotypes are publicly available (Yu and Ji, 2014). Though this assumption is reasonable for some studies (if one uses a public database, such as the 1000 genomes cohort, for the controls), it does limit the settings in which their technique can be applied.

Beyond just choosing high scoring SNPs, others have also looked at ways of estimating significance after choosing the SNPs of interest. This goal has been achieved by calculating the sensitivity of the allelic test statistic and applying the Laplace mechanism directly to it, or by performing similar procedures for *P*-values (Uhler *et al.*, 2013; Yu *et al.*, 2014).

## 2 Our contributions

We significantly improve upon the promising neighbor distance based mechanism for releasing top SNPs (which was introduced by Johnson and Shmatikov, 2013) and further refined by Yu *et al.* (2014) and Yu and Ji (2014). We introduce an adaptive threshold approach which overcomes accuracy issues arising from the fact that the neighbor mechanism might favor a different ordering than the true ordering given by the allelic test statistic. We then introduce a faster algorithm for calculating the neighbor distance (defined below) used in this method, making it tractable for large datasets. Moreover, unlike some previous approaches (Yu and Ji, 2014), our method ensures the privacy of individuals in both the case and control cohorts.

This algorithm works in three steps: (i) stating the problem as an optimization problem; (ii) solving a relaxation of this problem in constant time; and (iii) rounding the relaxed solution to a solution to the original problem.

We also show how to obtain accurate estimates of the allelic test statistic. In particular, we show that the input perturbation based method greatly improves accuracy over traditional output perturbation-based techniques when applied to the allelic test statistic (as opposed to some other statistics (Uhler *et al.*, 2013).

Finally, we apply our methods to real GWAS data, demonstrating both our greatly improved computational performance and accuracy compared with the state of the art.

## 3 Methods

### 3.1 Differential privacy

We begin with a data set $D=(d_{1},\ldots ,d_{n})\in D^{n}$ for which we want to calculate *f*(*D*) for some $f:D^{n}\to \Omega$, where Ω and D are both sets. For example, D might be the set of all possible genotypes. Often, however, *f*(*D*) releases private information about *d_i_* for some *i*. For example, if *D* is a set of patients with a given disease then *f*(*D*) may reveal the fact that *d_i_* is in *D*, and thus has the disease. In order to deal with this worry we want to release a perturbed version of *f*, let us call it *F*, that does not have the same privacy concerns. This idea is formalized using differential privacy (Dwork and Pottenger, 2013). We say that *D* and $D\prime =(d_{1}^{\prime },\ldots ,d_{n}^{\prime })$ are neighboring databases if they differ in exactly one entry (aka there is exactly one *i* such that $d_{i}\neq d_{i}\prime$). We then have the following definition.

Definition 1. A random function $F:D^{n}\to \Omega$ is ϵ-differentially private for some ϵ>0 if, for all neighboring databases D and D′ and all sets S⊆Ω, we have that

P(F(D)∈S)≤exp(ϵ)P(F(D′)∈S)

Intuitively, the above definition says that if *D* and D′ differ by one entry then *F*(*D*) and F(D′) are statistically hard to distinguish. This ensures that no individual has too large an affect on *F*(*D*), so no participant loses too much privacy. The parameter *ϵ* is a privacy parameter: the closer to 0 it is the more privacy is ensured, while the larger it is the weaker the privacy guarantee. Clearly this means we would like to set *ϵ* as small as possible, but unfortunately this comes at the cost of having less useful output. The problem of figuring out the correct *ϵ* to use is quite tricky (Hsu *et al.*, 2014).

Our goal is to find a differentially private *F* that closely approximates *f*. One of the simplest ways to do this is with what is known as the Laplacian mechanism (Dwork and Pottenger, 2013). Formally, if $\Omega \subseteq R^{k}$, we define the sensitivity of a function *f*, denoted Δf, to be equal to

$$\Delta f=\underset{D,D\prime \text{neighbors}}{\max}|f(D)-f(D\prime ){|}_{1}$$

More than that, let ${\text{Lap}}_{k}(\lambda )\in R^{k}$ be a random variable that returns a *k*-dimensional vector with probability density, $p_{k,\lambda }$, given by

$$p_{k,\lambda }(x)\propto \text{exp}\left(-\frac{|x{|}_{1}}{\lambda }\right)$$

We let $\text{Lap}(\lambda )={\text{Lap}}_{1}(\lambda )$. The Laplacian mechanism works by letting

$$F(D)=f(D)+{\text{Lap}}_{k}\left(\frac{\Delta f}{\epsilon }\right)$$

Theorem 1 (Dwork and Pottenger, 2013). If *F* is defined as above than *F* is ϵ-differentially private.

### 3.2 Allelic test statistic

The allelic test statistic is used to test for associations between SNPs and disease status. In order to define it, assume we have a case-control cohort. For a given SNP let *s*_0_, *s*_1_ and *s*_2_ be the number of individuals in the control population with 0, 1 or 2 copies of the minor allele, respectively. Similarly, let *r*_0_, *r*_1_ and *r*_2_ be the corresponding quantities for the case cohort, and *n*_0_, *n*_1_ and *n*_2_ be the same quantities over the entire study population. Let *S* be the number of cases, *R* the number of controls, and *N* the total number of participants. We assume that *R*, *S* and *N* are known.

The allelic test statistic is given by

$$Y(r_{0},r_{1},r_{2},s_{0},s_{1},s_{2})=\frac{2N{\left((2r_{0}+r_{1})S-(2s_{0}+s_{1})R\right)}^{2}}{RS(2n_{0}+n_{1})(n_{1}+2n_{2})}$$

Note that *Y* only depends on $x=2r_{0}+r_{1}$ and $y=2s_{0}+s_{1}$, so we can overload notation and let

$$Y(x,y)=\frac{2N{\left(xS-yR\right)}^{2}}{RS(x+y)(2N-x-y)}$$

### 3.3 Neighbor distance

Our goal is to pick the top *m_ret_* highest scoring SNPs (where *m_ret_* is a user chosen parameter). In order to do this we shall use the neighbor method. We begin by introducing some notation. For a set, *S*, we use |S| to denote the number of elements in *S*. Similarly, for a vector, *v*, let |v| denote the length of *v*. Moreover, for a given study cohort, denoted *D*, let $Y_{i}(D)$ be the allelic test statistic of the *i*th SNP.

The neighbor method for picking SNPs (Johnson and Shmatikov, 2013) starts with a user defined threshold, *ω*. All SNPs with an allelic score higher than *ω* are considered significant, while all others are considered not significant.

In order to understand how the neighbor method works, we must define the neighbor distance. The neighbor distance of a given SNP to the threshold *ω* is the minimum number of individuals whose genotypes need to be changed in our database to flip a given SNP from significant to not significant or vice versa—i.e. to say the minimum Hamming distance from our databases to a significant database if the SNP is not significant or vice versa. We can then use this distance measure to pick our SNPs in a differentially private manner, as shown in Algorithm 1.

Intuitively, the idea is that the neighbor distance is closely related to the allelic test statistic. For significant SNPs, the more strongly the SNP is associated to the disease, the larger the neighbor distance tends to be. Conversely, for SNPs that are not significant, a stronger association tends to correspond to a smaller neighbor distance. The neighbor mechanism harnesses this intuition by attempting to pick significant SNPs with large neighbor distances and SNPs that are not significant but have small neighbor distance.

Algorithm 1. The neighbor method for picking top *m_ret_* SNPs (Johnson and Shmatikov, 2013)

**Require:** Data set *D*, number of SNPs to return *m_ret_*, privacy value *ϵ*, and boundary *ω*.

**Ensure:** A list of *m_ret_* SNPs that is *ϵ*- differentially private.

**for** i=0,…,m **do**

**if** $Y_{i}(D)>\omega$ **then**

$d_{i}=\underset{D\prime }{\min}\;(\{|D-D\prime |:Y_{i}(D\prime )<\omega ,|D\prime |=|D|\})$

**else**

$d_{i}=1-\underset{D\prime }{\min}\;(\{|D-D\prime |:Y_{i}(D\prime )>\omega ,|D\prime |=|D|\})$

**end if**

**end for**

Let $\omega_{i}=\text{exp}{\left(\frac{\epsilon }{2m_{\text{ret}}}d\right)}_{i}$ for all *i*.

Choose *m_ret_* SNPs without replacement, where $\mathrm{Pr}(\text{Choose SNP}i)\propto \omega_{i}$.

**return** Chosen SNPS

### 3.4 Modified neighbor method

Though the neighbor method is much more accurate than other methods for most databases, it sometimes leads to incorrect results (Yu *et al.*, 2014). This is due to the fact that the ordering given by the allelic test score differs slightly from the ordering given by the neighbor distance. We show, however, that this can be dealt with by slightly changing Algorithm 1. Instead of picking a boundary *ω* beforehand, we use part of the privacy budget to choose an optimal boundary, *ω_dp_*, with the Laplacian mechanism (more details in the Supplementary Materials), then use the rest of the privacy budget to choose the SNPs. This algorithm is given in Algorithm 2.

Algorithm 2. Our modified neighbor method for picking top *m_ret_* SNPs

**Require:** Data set *D*, number of SNPs to return *m_ret_*, privacy values *ϵ*_1_ and *ϵ*_2_.

**Ensure:** A list of *m*_ret_ SNPs that is - $\epsilon_{1}+\epsilon_{2}$—differentially private.

Let *ω* be the mean score of the *m_ret_*th and $m_{\text{ret}}+1$-st highest scoring SNP.

Let *ω*_dp_ be an *ϵ*_1_-differentially private estimate of *ω* (use the Laplacian Mechanism).

**return** Chosen SNPS using Algorithm 1 with $\epsilon =\epsilon_{2}$ and boundary value *ω*_dp_.

Note, in practice, we pick *ϵ* and let $\epsilon_{1}=.1\epsilon$ and $\epsilon_{2}=.9\epsilon$. This is arbitrary, and it would be worthwhile looking at the trade-off between *ϵ*_1_ and *ϵ*_2_.

### 3.5 Quick neighbor distance

The major computational bottleneck of the neighbor method for picking high scoring SNPs has been the calculation of the neighbor distance. This bottleneck has led some to calculate approximate neighbor distances (Yu *et al.*, 2014) or use methods that leak information about the control cohort (Yu and Ji, 2014). We are able to overcome this bottleneck using Algorithm 3.

To help remedy the situation we introduce a new method for calculating the neighbor distance. Our method involves only a constant number of arithmetic operations per SNP. To understand our approach, assume we want to calculate the neighbor distance for a given SNP and a given threshold, *ω*. To simplify notation, let $\rho =(r_{0},r_{1},r_{2},s_{0},s_{1},s_{2})$. Note that the neighbor distance can be expressed as the solution to the following optimization problem:

$$\begin{array}{l} \underset{\rho^{\prime }\in Z^{6}}{\text{minimize}}\;\frac{1}{2}|\rho -\rho^{\prime }{|}_{1}\\ \text{subject to }\rho_{i}^{\prime }\geq 0,i=1,\ldots ,6\\ \;\rho_{0}^{\prime }+\rho_{1}^{\prime }+\rho_{2}^{\prime }=R,\;\rho_{3}^{\prime }+\rho_{4}^{\prime }+\rho_{5}^{\prime }=S\\ \;x^{\prime }=2\rho_{0}^{\prime }+\rho_{1}^{\prime },\;y^{\prime }=2\rho_{3}^{\prime }+\rho_{4}^{\prime }\\ \;u_{\omega }(\rho )(Y(x^{\prime },y^{\prime })-\omega )\leq 0 \end{array}$$

where $u_{\omega }(\rho )$ denotes the sign of Y(ρ)−ω. By removing the integrality constraints and projecting down onto two dimensions we get the following relaxation:

$$\begin{array}{l} \underset{x,y}{\text{minimize}}\;g(x,y)=g_{1}(x)+g_{2}(y)\\ \text{subject to }0\leq x\leq 2R;\;0\leq y\leq 2S\\ u_{\omega }(\rho )(Y(x,y)-\omega )\leq 0 \end{array}$$

where

$$g_{1}(x)=\{\begin{array}{ll} \frac{x-2r_{0}-r_{1}}{2} & 2R-r_{1}\geq x\geq 2r_{0}+r_{1}\\ \frac{2r_{0}+r_{1}-x}{2} & r_{1}\leq x\leq 2r_{0}+r_{1}\\ r_{2}+x-2(r_{0}+r_{2})-r_{1} & 2R\geq x\geq 2R-r_{1}\\ r_{0}+r_{1}-x & \text{otherwise} \end{array}$$

and

$$g_{2}(y)=\{\begin{array}{ll} \frac{y-2s_{0}-s_{1}}{2} & 2S-s_{1}\geq y\geq 2s_{0}+s_{1}\\ \frac{2s_{0}+s_{1}-y}{2} & s_{1}\leq y\leq 2s_{0}+s_{1}\\ s_{2}+y-2(s_{0}+s_{2})-s_{1} & 2S\geq y\geq 2S-s_{1}\\ s_{0}+s_{1}-y & \text{otherwise} \end{array}$$

See the Supplementary Materials for a more detailed derivation. We say that (*x*, *y*) is feasible if it satisfies the constraints for this relaxed problem.

Algorithm 3 first solves this relaxed problem by iterating over a small set of possible solutions (each of which can be found in constant time using the quadratic equation and some basic facts about convex optimization) then rounding to find a solution to the original problem. A proof of correctness as well as a few other details is given in the Supplementary Materials. Note that the algorithm involves *β*_1_ and *β*_2_, where

$$\beta_{1}(x)=\{\begin{array}{ll} \left\lceil g_{1}(x)\right\rceil +1 & \text{if}r_{1}=0\;\mathrm{and}\;x-2r_{0}-r_{1}\text{odd}\\ \left\lceil g_{1}(x)\right\rceil & \text{else} \end{array}$$

and

$$\beta_{2}(y)=\{\begin{array}{ll} \left\lceil g_{2}(y)\right\rceil +1 & \text{if}s_{1}=0\text{and}y-2s_{0}-s_{1}\text{odd}\\ \left\lceil g_{2}(y)\right\rceil & \text{else} \end{array}$$

Note that our algorithm assumes that $\omega \geq \frac{2N}{2N-1}$. This restriction, however, is not a problem, since in practice this corresponds to a rather large p-value (>.05 as long as *N* > 5). To accommodate this restriction, the only change we need to make to the neighbor method is to round *ω_dp_* up to $\frac{2N}{2N-1}$ if this condition is not met. It is also worth noting that this algorithm relies on being able to check, for a given *δ*, if there exists a feasible x,y∈Z with $\beta_{1}(x)+\beta_{2}(y)=\delta$. We show how to check these conditions in the Supplementary Materials.

Algorithm 3. Calculates the neighbor distance for SNPs in constant time

**Require:** $\rho =(r_{0},r_{1},r_{2},s_{0},s_{1},s_{2})$ with $\rho_{i}\geq 0$ for i=0,…,5; *N*, *R* and *S* defined as usual; and threshold $\omega \geq \frac{2N}{2N-1}$.

Let $g(x,y)=g_{1}(x)+g_{2}(y)$ be defined as in the text.

Let *C* denote the curve defined by

$$2N{\left(xS-yR\right)}^{2}=RS\omega (x+y)(2N-x-y)$$

Find the set *P* of all points p∈[0,2R]×[0,2S] on the curve *C* whose tangent line has slope in

$$\left\{1,2,\frac{1}{2}\right\}$$

Let *Q* be the set of all $p=(p_{0},p_{1})\in [0,2R]\times [0,2S]\cap C$ and either

$$p_{0}\in \{2(r_{0}+r_{2})+r_{1},2r_{0}+r_{1},r_{1},0,2R\}$$

or

$$p_{1}\in \{2(s_{0}+s_{2})+s_{1},2s_{0}+s_{1},s_{1},0,2S\}$$

$$\hat{g}=\min_{p\in P\cup Q}\left\lceil g(p)\right\rceil$$

**if** Y(ρ)<ω **then**

       **return** $\hat{g}$

**end if**

**for** $\delta \in \{\hat{g},\ldots ,\hat{g}+5\}$ **do**

     **if** exists feasible x,y∈Z with $\beta_{1}(x)+\beta_{2}(y)=\delta$ **then**

       **return** *δ*

     **end if**

**end for**

Theorem 2. Algorithm 3 returns the true neighbor distance for the specified SNP and involves only a constant number of arithmetic operations.

Proof. See the Supplementary Materials.

### 3.6 Input perturbation

In addition to returning high scoring SNPs, we want to return estimates of the allelic test statistic for those high scoring SNPs. In the past this has been achieved by applying the Laplacian mechanism to the output allelic test statistic (Yu *et al.*, 2014). Instead we apply the Laplacian mechanism to the inputs. The method works as follows: Let $x=2r_{0}+r_{1}$ and $y=2s_{0}+s_{1}$. Then we see that if x′ and y′ are the corresponding quantities for a neighboring database that |x−x′|+|y−y′|≤2. Therefore if we let

$$x_{\text{dp}}=x+\text{Lap}\left(\frac{2}{\epsilon }\right)$$

and

$$y_{\text{dp}}=y+\text{Lap}\left(\frac{2}{\epsilon }\right)$$

then $\left(x_{\text{dp}},y_{\text{dp}}\right)$ is a *ϵ*-differentially private estimate of (*x*, *y*). We can then estimate *Y* in a differentially private way using the equation

$$\frac{2N{\left(x_{\text{dp}}S-y_{\text{dp}}R\right)}^{2}}{RS(x_{\text{dp}}+y_{\text{dp}})(2N-x_{\text{dp}}-y_{\text{dp}})}$$

if the denominator is greater than 0, else outputting 0.

### 3.7 Measuring performance

In order to test our method we use the following standard measure of performance (Yu *et al.*, 2014). Let *A* be the top *m_ret_* scoring SNPs, and let *B* be the *m_ret_* SNPs returned by some differentially private algorithms. We than measure the utility of the algorithm by considering $\frac{\left|A\cap B\right|}{\left|A\right|}$. The closer to one this quantity is the better.

Note that one might also look at other measures of utility—after all, the difference between *m_ret_*th highest scoring SNP and the next highest scoring SNP may be small, and this measure does not consider that. We use this measure due to its simplicity, and because it has been used in previous works (Yu and Ji, 2014; Yu *et al.*, 2014).

### 3.8 Dataset

We test our methods on a rheumatoid arthritis dataset, NARAC-1, from Plenge *et al.* (2007). After quality control it contained 893 cases and 1244 controls. We removed all SNPs with minor allele frequency <0.05. We considered only SNPs that were successfully called for all individuals. This process resulted in a total of 62 441 SNPs to be considered.

## 4 Results

### 4.1 Comparison to the score and Laplacian-based methods

Our modified neighbor distance method outperforms both the Laplacian and score based methods (Yu *et al.*, 2014) for picking high scoring SNPs. In order to demonstrate this we run our algorithm and both the other algorithms for various *m_ret_* and *ϵ* to compare utility.

**Fig. 1:**
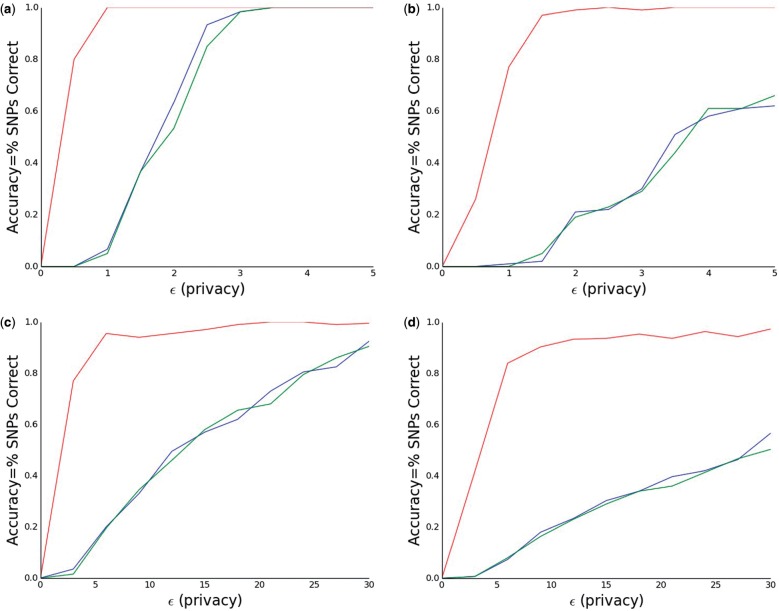
We measure the performance of our modified neighbor method for picking top SNPs (red) as well as the score based (blue) and Laplacian based (green) methods for *m*_ret_ (the number of SNPs being returned) equal to **(a)** 3, **(b)** 5, **(c)** 10 and **(d)** 15 for varying values of *ϵ*. For $m_{\text{ret}}=3,5$ we consider *ϵ* between 0 and 5, while in the other cases we consider *ϵ* between 0 and 30. We see that in all four graphs our method leads to the best performance by far. These results are averaged over 20 iterations

The results can be seen in Figure 1. We see that in all cases our modified neighbor method (red) outperforms the Laplacian (green) and score (blue) based methods by a large margin.

It is worth noting that the accuracy of the score and Laplacian based methods are fairly consistent with previous work (Yu and Ji, 2014). The most interesting difference is that the score and Laplacian based methods seem to perform more similarly in our experiments than in previous work (Uhler *et al.*, 2013; Yu and Ji, 2014; Yu *et al.*, 2014). This suggests that the relative performance of each method may be dataset dependent, depending on the number of SNPs, size of case and control cohorts, and the distribution of *P*-values (e.g. if there is a large gap between the score of the top *m_ret_* SNPs and the rest of the SNPs one might expect the above methods to be more accurate).

### 4.2 Comparison to the traditional neighbor method

**Fig. 2:**
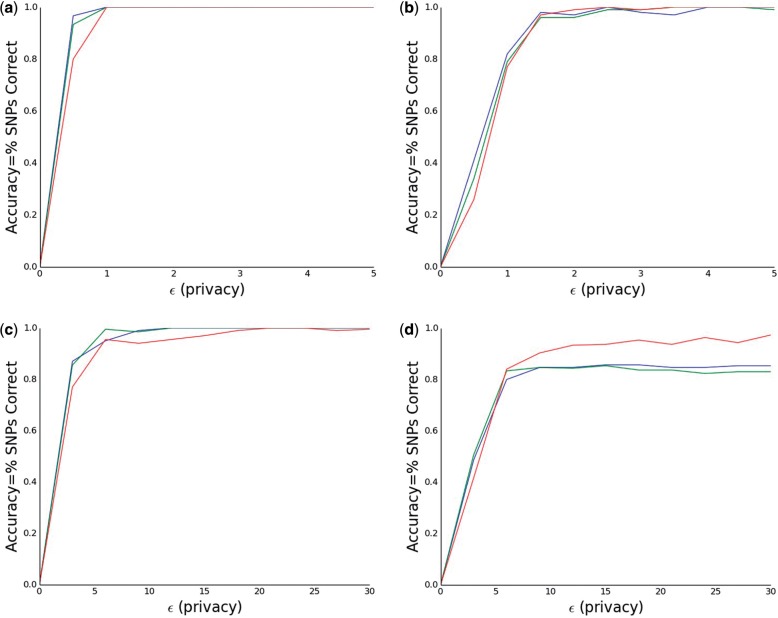
We measure the performance of our modified neighbor method for picking top SNPs (in red) as well as the traditional neighbor method with cutoffs corresponding to a Bonferroni corrected *P*-value of.05 (in green) and.01 (in blue) for *m*_ret_ (the number of SNPs being returned) equal to **(a)** 3, **(b)** 5, **(c)**10 and **(d)** 15 for varying values of *ϵ*. For $m_{\text{ret}}=3,5$ we consider *ϵ* between 0 and 5, while in the other cases we consider *ϵ* between 0 and 30. We see that in the first three cases the traditional method slightly outperforms ours. When *m*_ret_ = 15; however, the traditional methods can only get maximum utility around.85, where as ours can get utility arbitrarily close to 1. These results are averaged over 20 iterations

Our modified neighbor method also manages to overcome many of this issues present in the traditional neighbor method, which uses a predefined cutoff *ω*. To demonstrate this we compare our method to the traditional method. For the traditional method we use a cutoffs corresponding to a Bonferroni corrected *P*-values of.05 and.01 (Yu *et al.*, 2014). The results are pictured in Figure 2. When *m_ret_* = 15, we see that as *ϵ* increases the utility of our method (red) increases towards one, while the utility of the traditional methods (green for 0.05, blue for 0.01) seem to plateau around 0.85. This result demonstrates the advantages of using adaptively chosen boundaries, even if in some cases $\left(m_{\text{ret}}\in \{3,5,10\}\right)$ doing so leads to slightly decreased utility for small *ϵ*. Moreover, by changing the balance between *ϵ*_1_ and *ϵ*_2_, it seems plausible that even this slight decrease can be mostly overcome.

### 4.3 Runtime

Beyond overcoming utility issues, our method is able to improve runtime on real GWAS datasets by an order of magnitude. To demonstrate this, we look at how long it takes to calculate the neighbor distance for all SNPs (since this is the time consuming step). In the past others have had to implement approximate versions of the neighbor distance to make it run in a reasonable time (Yu *et al.*, 2014). We implemented a simple hill climbing algorithm similar to those used in previous works (Yu *et al.*, 2014). We then tested it for various values of *m_ret_* (see Table 1). We see that our method is much faster than the approximate method, taking only about 3 s in all cases to estimate the neighbor distances for all SNPs. Moreover, we see that the approximate method gives results that can greatly differ from our exact results, as demonstrated by the average error in the neighbor distance per SNP.

**Table:** **Table 1.** We demonstrate the runtime of our exact method as well as the approximate method for various numbers of SNPs as well as the average error per SNP that comes from using the approximate method

*m_ret_*	Our runtime	Approximate runtime	Approximate error
3	3.0 s	71.15 s	22.15
5	3.0 s	53.4 s	13.77
10	3.05 s	38.2 s	7.62
15	3.05 s	31.85 s	5.76

We see that in all cases the exact method is much faster than the approximate method. In addition, its runtime is fairly steady for all choices of *m_ret_*. These results are averaged over 20 trials.

### 4.4 Input versus output perturbation

**Fig. 3:**
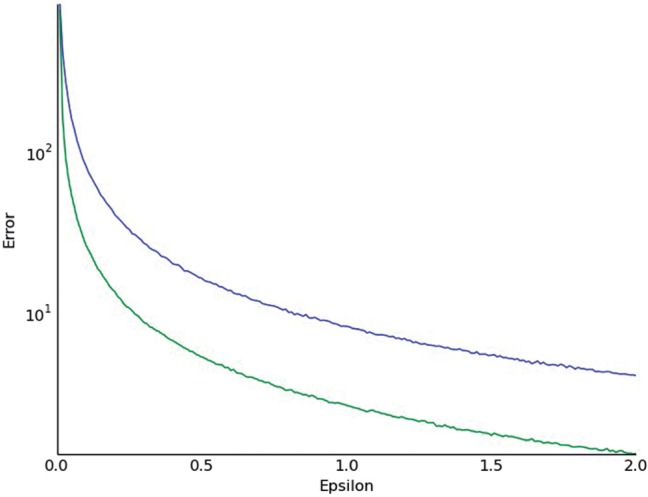
We compare the accuracy of output perturbation (blue) and input perturbation (green), tested on the 10 highest scoring SNPs. We see that the input perturbation approach greatly outperforms the standard output perturbation approach. This graph was averaged over 1000 runs, and the error is plotted on a log scale

Finally, we are able to show that our input perturbation method compares favorably to previous output perturbation based approaches. To see this, we looked at the average error of estimating the allelic test statistic on the top ten highest scoring SNPs for both input perturbation (green) and output perturbation (blue) (we considered the top 10 SNPs because we are usually only interested in the most significant SNPs—the performance is even more lopsided for arbitrary SNPs). We see that our input perturbation based approach greatly decreases the error compared with output perturbation based methods for *ϵ* between 0 and 2. It is worth noting that this result differs from the result of similar comparisons for the Pearson χ^2^-statistic, since in that case output perturbation seems preferable (Uhler *et al.*, 2013). This is likely due to the fact that we are adding noise to a 2 by 2 table of inputs, as opposed to a 2 by 3 table (Fig. 3).

## 5 Conclusion

The above work shows how to make differentially private GWAS much more realistic, both in terms of accuracy and run time. Though the tools of differential privacy have been around for years Mohan *et al.*, 2012, the biomedical community has been slow to adopt them (Dankar and El Emam, 2014). Though this delay is partially due to the limited knowledge about such approaches in the biomedical field, perhaps a bigger reason is that current techniques greatly reduce the utility of data and their analysis. In a field whose main concern is human health there is extra incentive to give the most accurate analysis possible—lives could be on the line.

Despite this concern, there are a few important areas where accurate differentially private methods might play a role. The most obvious one is when institutional or legal concerns prevent data from being published (Gilbert, 2008). When such limitations exist, it might be possible to release differentially private versions of the data under consideration instead. The other application where differential privacy might be useful is when untrusted users query a database. It is this situation that has motivated many of the previous works on differential privacy (Johnson and Shmatikov, 2013; Vinterbo *et al.*, 2012), and some of the only applications of data perturbation that have been implemented in real world systems (Lowe *et al.*, 2009; Murphy *et al.*, 2012). In a nutshell, the idea is that users who might want to use a large medical database to help design a study (e.g. to come up with hypothesis to test, find participants with certain traits for a study) or validate results can do so by asking queries about the database and getting differentially private answers to those queries. This approach allows researchers access to the database while minimizing privacy concerns. As an added bonus, since the queries are being used as a preliminary step, as opposed to being part of a rigorous analysis, there may be less worry about the ethical implications of returning inaccurate results. It is even possible that being able to make such queries will actually lead to more accurate results downstream.

## Supplementary Material

Supplementary Data
