# Supplementary material for: Realizing privacy preserving genome-wide association studies
Source: Bioinformatics. 2016 Jan 14;32(9):1293–300. doi: 10.1093/bioinformatics/btw009 (PMC4848404; doi:10.1093/bioinformatics/btw009)
Supplement: Supplementary Data [file supp_32_9_1293__index.html]

Realizing privacy preserving genome-wide association studies — Supplementary Data 

# Realizing privacy preserving genome-wide association studies

## Supplementary Data

files

- Supplementary Data - pdf file
