# Supplementary material for: Realizing privacy preserving genome-wide association studies
Source: Bioinformatics. 2016 Jan 14;32(9):1293–300. doi: 10.1093/bioinformatics/btw009 (PMC4848404; doi:10.1093/bioinformatics/btw009)
Supplement: Supplementary Data [file supp_btw009_SuppMaterial.pdf]

Supplementary Material for "Realizing Privacy Preserving Genome-wide Association Studies"

Sean Simmons and Bonnie Berger

January 5, 2016

Algorithm 1 Calculates the neighbor distance for SNPs in constant time

Require: $\rho = (r_0, r_1, r_2, s_0, s_1, s_2)$ with $\rho_i \geq 0$ for $i = 0, \ldots, 5$; N, R and S defined as usual; and threshold $\omega \geq \frac{2N}{2N-1}$.

Let $g(x, y) = g_1(x) + g_2(y)$ be defined as in the text.

Let C denote the curve defined by

$$2N(xS - yR)^2 = RS\omega(x + y)(2N - x - y)$$

Find the set P of all points $p \in [0, 2R] \times [0, 2S]$ on the curve C whose tangent line has slope in

$$\{1, 2, \frac{1}{2}\}$$

Let Q be the set of all $p = (p_0, p_1) \in [0, 2R] \times [0, 2S] \cap C$ and either

$$p_0 \in \{2(r_0 + r_2) + r_1, 2r_0 + r_1, r_1, 0, 2R\}$$

or

$$p_1 \in \{2(s_0 + s_2) + s_1, 2s_0 + s_1, s_1, 0, 2S\}$$

$\hat{g} = \min_{p \in P \cup Q} \lceil g(p) \rceil$

if $Y(\rho) < \omega$ **then**

  return $\hat{g}$

end if

for $\delta \in \{\hat{g}, \ldots, \hat{g} + 5\}$ **do**

  if exists feasible $x, y \in \mathbb{Z}$ with $\beta_1(x) + \beta_2(y) = \delta$ **then**

    return δ

  end if

end for

1 Introduction

In this document we prove that Algorithm 3 works as claimed. To do this, assume we are given a database D. We will show how to calculate the neighbor distance for a particular SNP. This allows us to specify our database with a tuple $\rho = (r_0, r_1, r_2, s_0, s_1, s_2)$ (see the main text for details). Let ω be the threshold we want to calculate the distance to.

Note that our proof relies on some basic concepts from convex analysis that can be found in most standard convex analysis books.

The first thing to notice is that the minimum Hamming distance from our database (with marginals given by $\rho = (r_0, r_1, r_2, s_0, s_1, s_2)$) to a database with marginals given by $\rho' = (r'_0, r'_1, r'_2, s'_0, s'_1, s'_2)$ is equal to $\frac{1}{2}|\rho - \rho'|_1$. This allows us to state the neighbor distance problem as the following optimization problem:

$$\begin{aligned} \underset{\rho' \in \mathbb{Z}^6}{\text{minimize}} \quad & \frac{1}{2}|\rho - \rho'|_1 \\ \text{subject to} \quad & \rho'_i \geq 0, i = 0, \ldots, 5 \\ & \rho'_0 + \rho'_1 + \rho'_2 = R,\ \rho'_3 + \rho'_4 + \rho'_5 = S \\ & x' = 2\rho'_0 + \rho'_1,\ y' = 2\rho'_3 + \rho'_4 \\ & u_\omega(\rho)(Y(x', y') - \omega) \leq 0 \end{aligned} \tag{1}$$

where $u_\omega(\rho)$ denotes the sign of $Y(\rho) - \omega$. To simplify this optimization problem, we begin by removing the integrality constraints, giving us:

$$\begin{aligned} \underset{\rho' \in \mathbb{R}^6}{\text{minimize}} \quad & \frac{1}{2}|\rho - \rho'|_1 \\ \text{subject to} \quad & \rho'_i \geq 0, i = 0, \ldots, 5 \\ & \rho'_0 + \rho'_1 + \rho'_2 = R,\ \rho'_3 + \rho'_4 + \rho'_5 = S \\ & x' = 2\rho'_0 + \rho'_1,\ y' = 2\rho'_3 + \rho'_4 \\ & u_\omega(\rho)(Y(x', y') - \omega) \leq 0 \end{aligned} \tag{2}$$

Since $Y(\rho')$ only depends on $x' = 2r'_0 + r'_1$ and $y' = 2s'_0 + s'_1$ we would like to reduce this to a two dimensional optimization problem. To do this reduction we need to consider $g(x, y) = g_1(x) + g_2(y)$, where

$$g_1(x) = \begin{cases} \frac{x - 2r_0 - r_1}{2} & \text{if } 2(r_0 + r_2) + r_1 \geq x \geq 2r_0 + r_1 \\ \frac{2r_0 + r_1 - x}{2} & \text{if } r_1 \leq x \leq 2r_0 + r_1 \\ r_2 + x - 2(r_0 + r_2) - r_1 & \text{if } 2R \geq x \geq 2(r_0 + r_2) + r_1 \\ r_0 + r_1 - x & \text{otherwise} \end{cases}$$

and

$$g_2(y) = \begin{cases} \frac{y - 2s_0 - s_1}{2} & \text{if } 2(s_0 + s_2) + s_1 \geq y \geq 2s_0 + s_1 \\ \frac{2s_0 + s_1 - y}{2} & \text{if } s_1 \leq y \leq 2s_0 + s_1 \\ s_2 + y - 2(s_0 + s_2) - s_1 & \text{if } 2S \geq y \geq 2(s_0 + s_2) + s_1 \\ s_0 + s_1 - y & \text{otherwise} \end{cases}$$

The importance of g is demonstrated by the following theorem:

Theorem 1. *Consider* $(x,y) \in [0,2R] \times [0,2S]$*, then*

$$2g(x,y) = \min_{\rho' \text{ feasible}, \; x=2\rho'_0+\rho'_1, \; y=2\rho'_3+\rho'_4} |\rho - \rho'|_1$$

Informally, $g(x,y)$ *is the minimum neighbor distance from* D *to a database with* $x = 2r'_0 + r'_1$ *and* $y = 2s'_0 + s'_1$*.*

Proof. It suffices to show that $g_1(x)$ is the minimum number of steps needed to reach a database with $x = 2r'_0 + r'_1$ and $g_2(y)$ is the minimum number of steps to reach a database with $y = 2s'_0 + s'_1$. We will prove it for $g_1(x)$, the argument for $g_2(y)$ being almost identical.

Consider the case that $x > 2r_0 + r_1$, the other case being similar. Since changing s'_i does not change $2r'_0 + r'_1$, we can assume that the s_i's stay fixed. First note that

$$2g_1(x) \geq \min_{\rho' \text{ feasible}, \; x=2\rho'_0+\rho'_1} |\rho - \rho'|_1$$

since it is achieved by increasing r'_0 and decreasing r'_2 until either $x = 2r'_0 + r'_1$, or until $r'_2 = 0$, in which case we decrease r'_1 and increase r'_0 until $x = 2r'_0 + r'_1$.

Moreover, this number of steps is clearly optimal. To see this, consider ρ' that achieves the above minimum, and note we can write

$$(r'_0, r'_1, r'_2) - (r_0, r_1, r_2) = u_1(1,0,-1) + u_2(1,-1,0)$$

for some $u_1, u_2 \in \mathbb{R}$. Then, by definition

$$2r_0 + r_1 + 2u_1 + u_2 = 2r'_0 + r'_1 = x$$

Meanwhile

$$|(r_0, r_1, r_2) - (r'_0, r'_1, r'_2)|_1 = |u_1| + |u_2| + |u_1 + u_2|$$

The fact that ρ' is positive is equivalent to $u_1 \leq r_2$, $u_2 \leq r_1$ and $u_1 + u_2 \geq -r_0$. Consider the case that $2u_1 < x - 2r_0 - r_1$. This implies $u_2 > 0$. Moreover, this implies $u_1 + u_2 > 0$. To see this, note that if $u_1 > 0$, this follows trivially from the fact that $u_2 > 0$. Otherwise, note that $-u_1 \geq 0$ so $u_1 + u_2 \geq 2u_1 + u_2 = x - 2r_0 - r_1 > 0$.

If $u_1 = r_2$, this gives us that $|(r_0, r_1, r_2) - (r'_0, r'_1, r'_2)|_1 = 2g_1(x)$. If, on the other hand, $u_1 < r_2$, choose any $\epsilon > 0$ so that $\epsilon < \min\{r_2 - u_1, u_1 + u_2, \frac{u_2}{2}\}$. Let $v_1 = u_1 + \epsilon$ and $v_2 = u_2 - 2\epsilon$. Then if

$$(r''_0, r''_1, r''_2) = (r_0, r_1, r_2) + v_1(1,0,-1) + v_2(1,-1,0)$$

we see that $r''_i \geq 0$ for $i = 0,1,2$, and that

$$|(r_0, r_1, r_2) - (r''_0, r''_1, r''_2)|_1 = |v_1(1,0,-1) + v_2(1,-1,0)|_1 = |u_1 + u_2 - \epsilon| + |u_2 - 2\epsilon| + |u_1 + \epsilon|$$

Since $u_1+u_2 > 0$ and $\epsilon < u_1+u_2$ we see that $|u_1+u_2-\epsilon| = |u_1+u_2|-\epsilon$. Similarly $|u_2-2\epsilon| = |u_2|-2\epsilon$. Finally, by the triangle inequality, $|u_1+\epsilon| \leq |u_1|+\epsilon$. Putting this together with the above we get that

$$|(r_0,r_1,r_2)-(r_0'',r_1'',r_2'')|_1 \leq |u_1+u_2|+|u_1|+|u_2|-2\epsilon < |u_1+u_2|+|u_1|+|u_2| = |(r_0,r_1,r_2)-(r_0',r_1',r_2')|_1$$

while $2r_0''+r_1'' = x$ and $r_i'' \geq 0$ for all i. This is a contradiction, so the theorem holds.

Applying similar arguments to the other cases gives us our result.

□

It is worth noting for later that this theorem has the following corollary. Let

$$\beta_1(x) = \begin{cases} \lceil g_1(x)\rceil + 1 & \text{if } r_1 = 0 \text{ and } x - 2r_0 - r_1 \text{ odd} \\ \lceil g_1(x)\rceil & \text{else} \end{cases}$$

and

$$\beta_2(y) = \begin{cases} \lceil g_2(y)\rceil + 1 & \text{if } s_1 = 0 \text{ and } y - 2s_0 - s_1 \text{ odd} \\ \lceil g_2(y)\rceil & \text{else} \end{cases}$$

then

Corollary 1. *Consider $(x,y) \in [0,2R]\times[0,2S]$ integral, then*

$$2(\beta_1(x)+\beta_2(y)) = \min_{\rho'\in\mathbb{Z}^6 \text{ feasible},\ x=2\rho_0'+\rho_1',\ y=2\rho_3'+\rho_4'} |\rho-\rho'|$$

This result implies that the optimal solution to our relaxed problem is equal to the optimal solution of

$$\begin{aligned} \underset{x,y}{\text{minimize}} \quad & g(x,y) = g_1(x)+g_2(y) \\ \text{subject to} \quad & 0 \leq x \leq 2R;\ 0 \leq y \leq 2S \\ & u_\omega(\rho)(Y(x,y)-\omega) \leq 0 \end{aligned} \tag{3}$$

while the solution to our initial integral problem is equal to the solution of

$$\begin{aligned} \underset{x,y\in\mathbb{Z}}{\text{minimize}} \quad & \beta_1(x)+\beta_2(y) \\ \text{subject to} \quad & 0 \leq x \leq 2R;\ 0 \leq y \leq 2S \\ & u_\omega(\rho)(Y(x,y)-\omega) \leq 0 \end{aligned} \tag{4}$$

It is important to note that

$$\{p|\frac{2N}{RS}(p_0S-p_1R)^2 \leq \omega(p_0+p_1)(2N-p_0-p_1)\}$$

is a convex set. More specifically, it is the convex hull of an ellipse. To see this, note that, since $\omega > 0$, it is easy to see that $2N(xS-yR)^2 \leq \omega RS(x+y)(2N-x-y)$ can be rewritten as $(x,y)Q(x,y)^T+q_1x+q_2y+q_3 \leq 0$ where Q is a positive

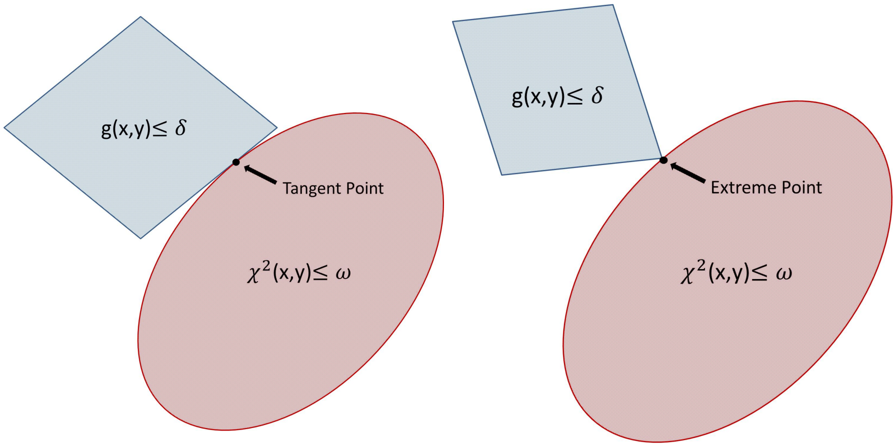

(a) Tangent Points (b) Extreme Points

Figure 1: Our algorithm for finding the solution, δ, of our relaxed optimization problem relies on the fact that there are only two possible types of solutions: (a) extreme point solutions and (b) tangent point solutions. Our algorithm finds all such extreme points and tangent points, and iterates over them to find the solution to our relaxed optimization problem.

semidefinite matrix. This implies $2N(xS - yR)^2 \leq \omega RS(x + y)(2N - x - y)$ is a (filled in) ellipse, and thus is convex.

In order to solve this relaxed problem we iterate through two sets of possible solutions, one corresponding to extreme points and one corresponding to tangent points (Figure 1). Using this solution, we are able to find the exact neighbor distance.

2 Proof for Significant SNPs

We first prove that Algorithm 3 works as advertised on significant SNPS (that is to say when $Y(\rho) > \omega$). We will first prove correctness, then prove it can be made to run in constant time.

Theorem 2. *Algorithm 3 returns the neighbor distance for significant SNPs.*

Proof. Assume we are looking at a significant SNP.

Assume that δ is the solution to the optimization problem in Equation 3, and that x', y' is the associated argmin. Then (x', y') must lay on the level set

$$D_\delta = \{(a, b) | 2R \geq a \geq 0, 2S \geq b \geq 0, g(a, b) = \delta\}$$

Note, however, that this set is the union of line segments, where each line segment has slope in $\{1, -1, 2, -2, .5, -.5\}$. Either (x', y') is an endpoint of one of these segments or is in the interior of one of them.

Let C be the curve defined by the equation:

$$\frac{2N}{RS}(x''S - y''R)^2 = \omega(x'' + y'')(2N - x'' - y'')$$

Note that $(x', y') \in C$. To see this, note g_1 and g_2 are strictly convex functions, so g is also strictly convex. Let $x = 2r_0 + r_1$ and $y = 2s_0 + s_1$. By definition $\frac{2N}{RS}(xS - yR)^2 > \omega(x + y)(2N - x - y)$ and $\frac{2N}{RS}(x'S - y'R)^2 \leq \omega(x' + y')(2N - x' - y')$. Therefore, by continuity, there exists a point, (a, b), on the line segment (endpoints included) connecting (x, y) and (x', y') so that

$$\frac{2N}{RS}(aS - bR)^2 = \omega(a + b)(2N - a - b)$$

Equivalently, $(a, b) \in C$. By the strict convexity of g it follows, since

$$g(x, y) = 0 < g(x', y')$$

that $g(a, b) \leq g(x', y')$, with equality if and only if $(a, b) = (x', y')$. Note, however, that (a, b) is feasible with respect to Equation 3, so by the optimality of (x', y') we get that $g(a, b) \geq g(x', y')$. Putting this all together, we get that $(a, b) = (x', y')$, so $(x', y') \in C$.

It is important to note that C is a smooth curve, so we can find its derivatives. If (x', y') is in the middle of a line segment $l \subset D_\delta$, we see that l must lie tangent to C, so (x', y') must be one of the points in C whose tangent line has slope in $\{1, -1, 2, -2, .5, -.5\}$.

Moreover, we can show that the slope of C at (x', y') is positive if $(x', y') \notin Q$, where Q is as defined in the algorithm. To see this, consider $b, c \in \mathbb{R}$ that are the slope and y-intercept of the line tangent to C at (x', y'). This implies $bx' + c - y' = 0$. Since $x' \geq 0$ and $y' \geq 0$ we see that $0 = bx' + c - y' \leq c - y' \leq c$. Note that equality holds if and only if $(x', y') = (0, 0)$. Similarly, since $2R \geq x'$ and $2S \geq y'$, the same argument implies that $0 \geq b2R + c - 2S$, with equality if and only if $(x', y') = (2R, 2S)$. If $(x', y') \notin Q$ this implies $(x', y') \neq (0, 0)$ and $(x', y') \neq (2R, 2S)$, so it follows that

$$b(0) + c - 0 < 0 < b(2R) + c - 2S$$

This implies the line which is tangent to C at (x', y') strictly separates the point $(0, 0)$ from $(2R, 2S)$. Note, however, that $(0, 0) \in C$ and $(2R, 2S) \in C$. Since C is the boundary of a convex shape (the ellipse defined by $\frac{2N}{RS}(x''S - y''R)^2 = \omega(x'' + y'')(2N - x'' - y'')$), we know from basic convex analysis that any line that is tangent to C can not strictly separate two points in C. Thus we have reached a contradiction, so it must be that the slope of the tangent line to C at (x', y') is positive if $(x', y') \notin Q$.

Putting the above together, (x', y') must either be in the set Q or must be one of the points in C whose tangent line has slope in $\{1, .5, 2\}$—the set we denoted by P in Algorithm 3.

Therefore either $(x', y') \in P \cup Q$ or (x', y') is the endpoint of some segment in D_δ. If it is an endpoint, however, it must be that $(x', y') \in Q$. Therefore, it

must be that $(x', y') \in P \cup Q$, so the quantity $\hat{g}$ calculated by Algorithm 3 is equal to $\lceil g(x', y') \rceil$.

We next want to show that there exists an integer pair (x, y) with $0 \leq x \leq 2R$, $0 \leq y \leq 2S$, $|x - x'| < 1$, $|y - y'| < 1$, and

$$\frac{2N}{RS}(xS - yR)^2 \leq \omega(x + y)(2N - x - y)$$

We will prove this fact in the case that $x'S - y'R > 0$ and $R \leq S$, the other cases being similar. Form (x, y) by rounding x' down and y' up. If $xS - yR \geq 0$ it must be that (x, y) is contained in the triangle whose vertices are (x', y'), $(0, 0)$ and $(2R, 2S)$. Note if $p = (p_0, p_1)$ is any one of these vertices then

$$\frac{2N}{RS}(p_0S - p_1R)^2 \leq \omega(p_0 + p_1)(2N - p_0 - p_1)$$

so it follows by convexity that

$$\frac{2N}{RS}(xS - yR)^2 \leq \omega(x + y)(2N - x - y)$$

Therefore consider the case that $xS - yR < 0$. Let E be the set of all $p \in [0, 2R] \times [0, 2S]$ with

$$\frac{2N}{RS}(p_0S - p_1R)^2 \leq \omega(p_0 + p_1)(2N - p_0 - p_1)$$

Note that, since $\omega > \frac{2N}{N-1}$, $(0, 2)$ and $(2R, 2S - 2)$ must be in the feasible region, E. Therefore, since $(0, 2)$, $(0, 0)$, $(2R, 2S-2)$ and $(2R, 2S)$ are all in E, by convexity the parallelogram formed by them, denoted by W, must also be in E. Let F be the closure of $[0, 2R] \times [0, 2S] - W$. Note $(x', y') \in F$, since (x', y') is on the boundary of E. Similarly if $(x, y) \notin E$ then $(x, y) \in [0, 2R] \times [0, 2S] - W \subset F$.

Note, however, that F has two components, E_0 and E_1, where $p_0S - p_1R > 0$ if $(p_0, p_1) \in E_0$ and $p_0S - p_1R < 0$ if $(p_0, p_1) \in E_1$. This fact implies $(x', y') \in E_0$ and $(x, y) \in E_1$. Note, however, that the L_2 distance between any point in E_1 and any point in E_0 is at least $\sqrt{2}$. By definition, however, $|(x, y) - (x', y')|_2 < \sqrt{2}$. This result is a contradiction, so it must be that $(x, y) \in E$, as desired.

Note $g(x', y') \leq g(x, y) \leq g(x', y') + 2 \leq \hat{g} + 2$. Furthermore,

$$\beta_1(x) + \beta_2(y) < g(x, y) + 4 \leq g(x', y') + 6$$

Finally, note that if $(a, b) \in E$ with $(a, b) \in \mathbb{Z}^2$ then

$$g(x', y') \leq g(a, b) \leq \beta_1(a) + \beta_2(b)$$

This fact implies that the neighbor distance is somewhere between $\hat{g}$ and $\hat{g} + 6$. Therefore Algorithm 3 returns the neighbor distance.

□

Theorem 3. *Algorithm 3 requires a constant number of arithmetic operations.*

Proof. First, note that P can be generated in a constant number of operations using ideas from basic calculus.

We can also generate Q in constant time. This can be done by first iterating over all $p_0 \in \{2(r_0 + r_2) + r_1, 2r_0 + r_1, r_1, 0, 2R\}$ and, for each of them, use the quadratic equation to find p_1 so that $Y(p_0, p_1) = \omega$, then doing something similar for each $p_1 \in \{2(s_0 + s_2) + s_1, 2s_0 + s_1, s_1, 0, 2S\}$.

Since $|P \cup Q| \leq 26$ and g takes constant time to calculate it follows that calculating $\hat{g}$ takes a constant number of arithmetic operations.

All that remains to prove is that, for a given integer δ, deciding if there is an integer pair $(x, y) \in [0, 2R] \times [0, 2S]$ with $\frac{2N}{RS}(xS - yR)^2 \leq \omega(x+y)(2N - x - y)$ and

$$\beta_1(x) + \beta_2(y) = \delta$$

can be done in a constant number of arithmetic operations. To see this fact, consider the case when $r_1 > 0$ and $s_1 > 0$, the other cases being similar. Note we can write

$$\beta_1(x) = \begin{cases} \frac{x-2r_0-r_1}{2} & \text{if } 2(r_0+r_2)+r_1 \geq x \geq 2r_0+r_1, \text{ and } x-2r_0-r_1 \text{ even} \\ \frac{x-2r_0-r_1+1}{2} & \text{if } 2(r_0+r_2)+r_1 \geq x \geq 2r_0+r_1,\ x-2r_0-r_1 \text{ odd} \\ \frac{2r_0+r_1-x}{2} & \text{if } r_1 \leq x \leq 2r_0+r_1,\ x-2r_0-r_1 \text{ even} \\ \frac{2r_0+r_1-x+1}{2} & \text{if } r_1 \leq x \leq 2r_0+r_1,\ x-2r_0-r_1 \text{ odd} \\ r_2 + x - 2(r_0+r_2) - r_1 & \text{if } 2R \geq x \geq 2(r_0+r_2)+r_1,\ x \text{ odd or even} \\ r_0 + r_1 - x & \text{otherwise, } x \text{ odd or even} \end{cases}$$

$$\beta_2(y) = \begin{cases} \frac{y-2s_0-s_1}{2} & \text{if } 2(s_0+s_2)+s_1 \geq y \geq 2s_0+s_1, \text{ and } y-2s_0-s_1 \text{ even} \\ \frac{y-2s_0-s_1+1}{2} & \text{if } 2(s_0+s_2)+s_1 \geq y \geq 2s_0+s_1,\ y-2s_0-s_1 \text{ odd} \\ \frac{2s_0+s_1-y}{2} & \text{if } s_1 \leq y \leq 2s_0+s_1,\ y-2s_0-s_1 \text{ even} \\ \frac{2s_0+s_1-y+1}{2} & \text{if } s_1 \leq y \leq 2s_0+s_1,\ y-2s_0-s_1 \text{ odd} \\ s_2 + y - 2(s_0+s_2) - s_1 & \text{if } 2S \geq y \geq 2(s_0+s_2)+s_1,\ y \text{ odd or even} \\ s_0 + s_1 - y & \text{otherwise, } y \text{ odd or even} \end{cases}$$

The above tells us we can define $c_0, \cdots, c_7$ so $c_i \in \{0, 1\}$, intervals $U_0, \ldots, U_7$ in $\mathbb{R}$, and rational $v_0, \cdots, v_7, d_0, \cdots, d_7 \in \mathbb{Q}$ so that

$$\beta_1(x) = v_i x + d_i \text{ iff } x \in U_i, \text{ and } x \equiv c_i \bmod 2$$

Note that, even though β_1 only has six cases, we need eight equations to define it instead of six. This is because the last two cases defining β_1 correspond to two equations each (one for x odd, one for x even).

Similarly we can define $c'_0, \cdots, c'_7$ so $c'_i \in \{0, 1\}$, intervals $U'_0, \ldots, U'_7$ in $\mathbb{R}$, and rational $v'_0, \cdots, v'_7, d'_0, \cdots, d'_7 \in \mathbb{Q}$ so that

$$\beta_2(y) = v'_i y + d'_i \text{ iff } y \in U'_i, \text{ and } y \equiv c'_i \bmod 2$$

Thus $\beta_1(x) + \beta_2(y) = \delta$ if and only if, for some $i, j \in \{0, \ldots, 7\}$, we have that

$$v_i x + v'_j y + d_i + d'_j = \delta$$

subject to $x \in U_i$, $y \in U'_j$, $x \equiv c_i \bmod 2$ and $y \equiv c'_i \bmod 2$. For a given i and j we can easily check if such an integral x and y exist.

More explicitly, let $\alpha = (\alpha_0, \alpha_1) = (1, \frac{-v_i}{v'_j})$ and $\beta = (\beta_0, \beta_1) = (0, \frac{\delta - d_i - d'_j}{v'_j})$, then $\alpha t + \beta$ is a parameterization of

$$\{(x, y) | v_i x + v'_j y + d_i + d'_j = \delta\}$$

Note that a vector with integer entries is said to be integral. Since $v_i, v'_j \in \{1, \frac{1}{2}\}$ either α has integer entries, or it has some entry that is not an integer but is an integer divided by two. Since $\alpha_0 = 1$ we see that if $\alpha t + \beta$ is integral then t must be an integer. Moreover, we see that $\alpha t + \beta$ is integral if and only if $\alpha(t + 4) + \beta$ is, and if both are integral they equal one another mod 2. We can easily find the interval $[\gamma_1, \gamma_2]$ so that $\alpha t + \beta \in U_i \times U'_j$ if and only if $t \in [\gamma_1, \gamma_2]$. Similarly, using the quadratic equation we can find the interval $[f_1, f_2]$ so that $Y(\alpha t + \beta) \leq \omega$ if and only if $t \in [f_1, f_2]$. If we let $[r, s]$ be the intersection of these two intervals it follows trivially that, if $(x, y) = \alpha t + \beta$, then $\frac{2N}{RS}(xS - yR)^2 \leq \omega(x + y)(2N - x - y)$ and

$$\beta_1(x) + \beta_2(y) = \delta$$

if and only if $t \in [r, s]$. It is easy to check in constant time if there is a $t_0 \in [r, s]$ such that

$$\alpha t_0 + \beta \equiv (c_1, c_2) \bmod 2$$

This follows from the fact that if t is a solution so is $t + 4$; hence there is such a t_0 iff there exists such a $t_0 \in \{\lceil r \rceil, \ldots, \lceil r \rceil + 4\}$ that also satisfies the conditions required. In conclusion, we can check in constant time if, given integral δ, there is an integer $(x, y) \in [0, 2R] \times [0, 2S]$ with $\frac{2N}{RS}(xS - yR)^2 \leq \omega(x + y)(2N - x - y)$ and

$$\beta_1(x) + \beta_2(y) = \delta$$

by iterating over all choices of i and j. Therefore this algorithm runs in constant time. □

3 Proof for Non-Significant SNPs

Note that an almost identical argument to the above can be used for non-significant SNPs if we are willing to do a similar rounding procedure. In order to make the algorithm more efficient, however, we show that a much simpler rounding procedure works.

Assume we are given a threshold ω and a database with $\rho = (r_0, r_1, r_2, s_0, s_1, s_2)$, where $\rho_i \geq 0$ for all i and $Y(\rho) < \omega$. Then we can calculate

$$\mu(\rho, \{v | Y(v) \geq \omega\})$$

in constant time (where, for a given set S, $\mu(v, S) = \min_{s \in S} \frac{1}{2}|v - s|_1$). In order to perform this calculation, consider Equation 3. We note that

Definition 1. *A real valued function f is quasi-convex if, for every $a \in \mathbb{R}$, $f^{-1}((-\infty, a))$ is a convex set.*

Lemma 1. *$Y : \mathbb{R}^2 \to \mathbb{R}$ is continuous and quasi-convex on $[0, 2R] \times [0, 2S]$.*

Proof. We know that Y is continuous from the previous section (assuming we let $Y(0,0) = Y(2R, 2S) = 0$)

To see that it is quasiconvex, note $Y(x,y) \leq a$ if and only if $2N(xS - yR)^2 \leq aRS(x+y)(2N - x - y)$. If $a < 0$ this is an empty set, if $a = 0$ this is a line. If, on the other hand, $a > 0$, it is easy to see that $2N(xS - yR)^2 \leq aRS(x+y)(2N-x-y)$ can be rewritten as $(x,y)Q(x,y)^T + q_1 x + q_2 y + q_3 \leq 0$ where Q is positive semidefinite. This fact implies $2N(xS - yR)^2 \leq aRS(x+y)(2N-x-y)$ is a (filled in) ellipse, and thus is convex. □

Theorem 4. *Algorithm 3 is correct on non-significant SNPs and runs in constant time.*

Proof. The proof that it runs in constant time is the same as for significant SNPs, so we need only prove correctness.

To see this, assume that δ is the solution to Equation 3. Let

$$C_\delta = \{(x,y) | g(x,y) \leq \delta\}$$

Then C_δ is a convex polygon. Furthermore, by definition of δ, we see that

$$\omega = \max_{(x,y) \in C_\delta} Y(x,y)$$

Since Y is quasi-convex and C_δ is a convex polygon, these facts imply that there is some extreme point of C_δ, let us call it (x,y), so that $Y(x,y) = \omega$. Since (x,y) is an extreme point of C_δ it must be that

$$x \in \{2(r_0 + r_2) + r_1, 2r_0 + r_1, r_1, 0, 2R\}$$

or

$$y \in \{2(s_0 + s_2) + s_1, 2s_0 + s_1, s_1, 0, 2S\}$$

so $(x,y) \in P$ by definition. Thus

$$\delta = \min_{p \in P} g(p) = \min_{p \in P \cup Q} g(p)$$

Assume

$$x \in \{2(r_0 + r_2) + r_1, 2r_0 + r_1, r_1, 0, 2R\}$$

the other case being similar. Then note that this assumption implies $g_1(x) = \beta_1(x)$ by definition.

We therefore consider y. By the proof of Theorem 1 we know that there exists $s'_0, s'_1, s'_2 \geq 0$ so that $s'_0 + s'_1 + s'_2 = S$, $2s'_0 + s'_1 = y$ and

$$2g_2(y) = |(s_0, s_1, s_2) - (s'_0, s'_1, s'_2)|_1$$

Moreover, the proof implies that either $s'_0 = 0$, $s'_2 = 0$ or $s'_1 = s_1$. Assume that $s'_1 = s_1$, the other cases being similar. Then let us define

$$u = (u_0, u_1, u_2) = (\lceil s'_0 \rceil, s'_1, \lfloor s'_2 \rfloor)$$

and

$$v = (v_0, v_1, v_2) = (\lfloor s'_0 \rfloor, s'_1, \lceil s'_2 \rceil)$$

Note that $2u_0 + u_1 \geq 2s'_0 + s'_1 \geq 2v_0 + v_1$ were $y = 2s'_0 + s'_1$, so by quasi-convexity either $\omega \leq Y(x, y) \leq Y(x, 2u_0 + u_1)$ or $\omega \leq Y(x, y) \leq Y(x, 2v_0 + v_1)$. Assume $\omega \leq Y(x, y) \leq Y(x, 2u_0 + u_1)$, the other case being identical. Note by construction $\frac{1}{2}|u - (s'_0, s'_1, s'_2)| < 1$ so

$$\frac{1}{2}|u - (s_0, s_1, s_2)| \leq \frac{1}{2}(|u - (s'_0, s'_1, s'_2)| + |(s_0, s_1, s_2) - (s'_0, s'_1, s'_2)|) < 1 + g_2(y)$$

Note, however, that $\frac{1}{2}|u - (s_0, s_1, s_2)|$ is an integer so it must be that

$$\frac{1}{2}|u - (s_0, s_1, s_2)| \leq \lceil g_2(y) \rceil$$

Note also that $(r_0, r_1, r_2, u_0, u_1, u_2)$ is feasible given the constraints in Equation 1 with associated score bounded above by

$$g_1(x) + \lceil g_2(y) \rceil = \lceil g_1(x) + g_2(y) \rceil = \lceil \delta \rceil$$

so the optimal value for Equation 1 is bounded above by $\lceil \delta \rceil$.

At the same time, since δ is the solution to Equation 3 we see that the solution to Equation 1 must be greater than or equal to δ. Since it must also be an integer it follows that the optimal solution must be greater than or equal to $\lceil \delta \rceil$. Putting this all together proves that the optimal solution to Equation 1 equals

$$\lceil \delta \rceil = \lceil \min_{p \in P \cup Q} g(p) \rceil = \hat{g}$$

and thus Algorithm 3 is correct for non-significant SNPs. □

Putting this together we get that Algorithm 3 is indeed correct, and runs in constant time per SNP!

4 Choosing ω_{dp}

Our algorithm relies on choosing a boundary, ω_{dp}, in a privacy preserving way. The main idea is that we would like to find a boundary that separates the top m_{ret} scoring SNPs from the rest of the SNPs. This can be achieved using the boundary ω, where ω is the mean score of the m_{ret}th and $m_{ret} + 1$-st highest scoring SNP. This choice of boundary does not preserve privacy, so we need to get a privacy preserving estimate of ω. Note, however, that the sensitivity of ω is equal to the sensitivity of the allelic test statistic. Since this sensitivity was calculated by [Yu *et al.*, 2014], we can apply the Laplacian mechanism to get a privacy preserving estimate of ω, namely ω_{dp}.

5 Overview of Score Method

Here we give an overview of the score method as presented in [Yu *et al.*, 2014].

Let Δ equal the maximum value in the set

$$\{\frac{8N^2S}{R(2S+3)(2S+1)}, \frac{4N^2((2R^2-1)(2S-1)-1)}{RS(2R+1)(2R-1)(2S+1)}, \frac{8N^2R}{S(2R+3)(2R+1)}, \frac{4N^2((2S^2-1)(2R-1)-1)}{RS(2S+1)(2S-1)(2R+1)}\}$$

This allows us to describe the score based method for picking high scoring SNPs:

Algorithm 2 The score based method for choosing SNPs ([Yu *et al.*, 2014])

Require: The genotypes of N individuals at M SNPs, with R cases and S controls, the number of SNPs to return (m_{ret}), and privacy parameter (ϵ)
  Let χ_i^2 be the allelic test statistic at SNP i, $i = 1, \ldots, m$
  Let $ret = \{\}$ be an empty set
  for $i = 1, \ldots, m_{ret}$ **do**
    Pick $j \in \{1, \cdots, m\}$, $j \notin ret$, where

$$P(\text{pick } j) \propto exp(\frac{\epsilon\chi_j^2}{2\Delta m_{ret}})$$

    Add j to ret
  end for
    return ret;

6 Overview of Laplacian Method

Here we give an overview of the Laplacian method as presented in [Yu *et al.*, 2014]. Let Δ be as in the previous section, than we can describe the Laplacian based method for picking high scoring SNPs:

Algorithm 3 The Laplacian based method for choosing SNPs ([Yu *et al.*, 2014])

Require: The genotypes of N individuals at M SNPs, with R cases and S controls, the number of SNPs to return (m_{ret}), and privacy parameter (ϵ)
  Let χ_i^2 be the allelic test statistic at SNP i, $i = 1, \ldots, m$
  Let $s_i = \chi_i^2 + Lap(0, \frac{2m_{ret}\Delta}{\epsilon})$
    return The m_{ret} SNPs with largest values of s_i

7 Weaker Definitions of Privacy

In the past, some works [Yu and Ji, 2014] have looked at weaker definitions of privacy. In particular, they have assumed that we are only interested in protecting the genomic data from the case cohort and not the control cohort. Previous work [Uhler *et al.*, 2013] has suggested that these two scenarios lead to similar performance in terms of accuracy when applying the obvious generalizations of existing methods. We show here that, by slightly tweaking these methods, better performance can be achieved in certain cases.

We consider two different scenarios in this chapter. In scenario 1 (the scenario considered elsewhere in the paper) we assume that all genomic data and phenotypic data is private– that is to say that only R, S and N are known. In scenario 2 we don't try to hide information about the control cohort and instead only try to hide the case cohort– that is to say that s_0, s_1 and s_2 are also known. This scenario has been investigated in previous work [Yu and Ji, 2014, Yu *et al.*, 2014].

In the past estimates of the allelic test statistic have been achieved by applying the Laplacian mechanism to the output of the allelic test statistic. It turns out, however, that in practice this choice is not optimal. Here we show how to achieve improved output perturbation when in scenario 2 by perturbing the square root of the allelic test statistic, and apply this to the Laplacian based mechanism for picking high scoring SNPs (Algorithm 4). This demonstrates that, by slightly changing the algorithms being used, scenario 2 can achieve greater utility than scenario 1.

Algorithm 4 The Laplacian method for picking top m_{ret} SNPs

---

Require: Data set D, number of SNPs to return m_{ret}, privacy value ϵ, and score function q that takes in a SNP and a dataset and returns a score.
Ensure: A list of m_{ret} SNPs

Let $\Delta q = \max_{i=1,\cdots,m, D\sim D'} |q(i,D) - q(i,D')|$.

Let $s_i = q(i,D) + Lap(0, \frac{2m_{ret}\Delta q}{\epsilon})$ for all i.

return The m_{ret} SNPs with highest s_i

7.1 Calculating Sensitivity

In order to apply output perturbation to a function q we need to calculate q's sensitivity. We discuss this below for both the allelic test statistic and its square root.

In scenario 1, if we choose q to be the allelic test statistic then we can use the sensitivity calculated by Yu et al.[Yu *et al.*, 2014].

In scenario 2, if we choose q to be $\sqrt{Y}$, we can let

$$f(x) = \sqrt{\frac{2N}{RS(2s_0 + s_1 + x)(2N - s_1 - 2s_0 - x)}}\,|xS - (2s_0 + s_1)R|$$

Then $f(2r_0 + r_1) = \sqrt{Y}$. This implies

$$\Delta\sqrt{Y} = \max_{x,y \in \{0,\cdots,2R\}, |x-y| \leq 2} |f(x) - f(y)|$$

We do not, however, have to iterate over all such x and y. Instead, let Ω be the set of all solutions to $f''(x) = 0$ such that $0 < x < 2R$, unioned with$\{0, 2R, \frac{(2s_0+s_1)R}{S}\}$. Let $\Omega' = \{\lfloor\omega\rfloor | \omega \in \Omega\} \cup \{\lceil\omega\rceil | \omega \in \Omega\}$. Then it follows from basic calculus based that

$$\Delta\sqrt{Y} = \max_{x \in \Omega', y \in \{x+2, x-2, x-1, x+1\}, 0 \leq y \leq 2R, |x-y| \leq 2} |f(x) - f(y)|$$

Note that Ω contains at most six elements and can be found easily (it comes down to finding the roots of a degree four polynomial), and as such it is easy to calculate $\max_{x \in \Omega', y \in \{0,\cdots,2R\}, |x-y| \leq 2} |f(x) - f(y)|$ in constant time.

In the remaining cases we estimate the sensitivity by brute force over all possible values of $2r_0 + r_1$ and $2s_0 + s_1$ under the corresponding scenarios. We did not bother deriving the sensitivity analytically because these methods are shown to be less than optimal in the following sections.

7.2 Output Perturbation

We first consider how Laplacian based output perturbation affects accuracy. Previous methods have worked by estimating the sensitivity of the allelic test statistic and applying the Laplacian mechanism to it directly [Uhler *et al.*, 2013]. Since we are now able to calculate the sensitivity of all of the above methods, we want to test how well each of them performs. In order to do this comparison we ran each method on our GWAS data and compared the errors, where the error is measured by L_1 error. We shall see that, in scenarios 2, our square root based approach outperforms previous approaches.

7.2.1 Scenario 2

We apply the methods to scenario 2. The results are pictured in Figure 2. In Figure 2(a) we choose 1000 SNPs at random and apply the Laplacian mechanism both directly to the allelic test statistic (the green curve) and to the square root of the allelic test statistic and square the result (the blue curve). We see that using the square root is the preferable method.

We also consider how the two approaches compare on high scoring SNPs (since we are most interested in these SNPs). In order to test this we measured the error on the 10 highest scoring SNPs, the result being pictured in Figure 2(b). We see that in this case applying the Laplace mechanism to the square root still outperforms the standard approach by a large amount.

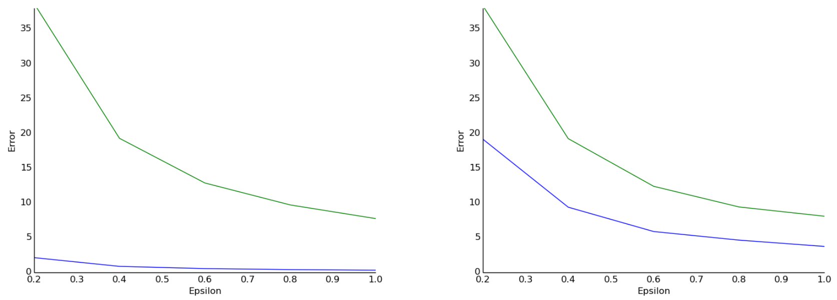

(a) All SNPs (b) 10 Highest Scoring SNPs

Figure 2: Comparing two forms of output perturbation in scenario 2– the first coming from applying the Laplace mechanism directly to the allelic test statistic (green), the other applying it to the square root of the allelic test statistic then squaring the result (blue), comparing the L_1 error on the y axis with ϵ on the x. We first apply it to 1000 random SNPs (a), then to the top ten highest scoring SNPs (b). We see that in both cases applying the statistic to the square root outperforms the standard approach.

7.2.2 Across Scenario Comparison

We now want to compare across scenarios. In particular we wonder if choosing not to hide genomic information can lead to utility gains. To test this scenario we took the best approach under each of the above regimes and compared the results (e.g., for scenario 2 we used the square root approach, for scenario 1 added the noise directly to the statistic). The results are plotted in Figure 3. In Figure 3(a) we plot the result of applying output perturbation to 1000 random SNPs under scenario 1 (red curve) and scenario 2 (blue curve), while in Figure 3(b) we do the same with the top ten highest scoring SNPs. In both cases we see that scenario 2 give us huge gains in utility.

7.3 Picking Top SNPs with the Laplacian Mechanism

We can apply the above output perturbation method to picking high scoring SNPs using Algorithm 4. The first set up will be with q equal to the allelic test statistic in scenario 1. We will use the square root of the allelic test statistic in scenario 2. The motivation for considering these is that, as we saw above, these are the two output perturbation approaches that perform best on GWAS data.

The results of this test are pictured in Figure 4. We apply both methods to the RA dataset, using $m_{ret} \in \{3, 5, 10, 15\}$ for figures 4(a), 4(b), 4(c), and 4(d) respectively. In each of the figures the blue curve compares the utility with ϵ for scenario 1 and the green curve compares the utility with ϵ for scenario 2. We see that, in all three cases, scenario 2 performs the best, greatly outperforming scenario 1, which is the set up previously consider in the literature

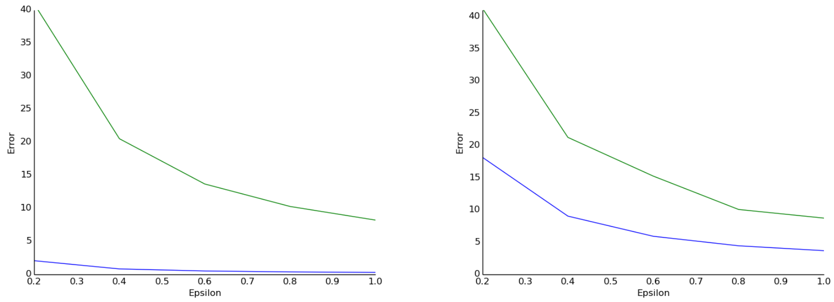

(a) All SNPs

(b) 10 Highest Scoring SNPs

Figure 3: Comparing the output perturbation of the allelic test statistic for scenarios 1 and 2, comparing the L_1 error on the y axis with ϵ on the x. In scenarios 2 (the blue curve) we add the noise to the square root then square the result, where as for scenario 1 (the green curve) we apply the Laplacian mechanism directly to the test statistic (this choice is motivated by the previous figures). We first apply it to 1000 random SNPs (a), then to the top ten highest scoring SNPs (b). We see that in scenario 2 we require much less noise than scenario 1.

[Uhler *et al.*, 2013].

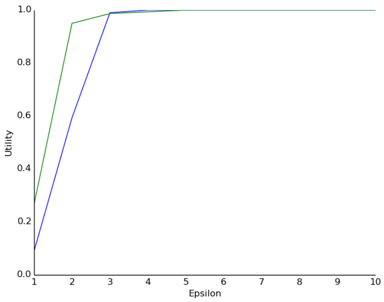

(a) $m_{ret} = 3$

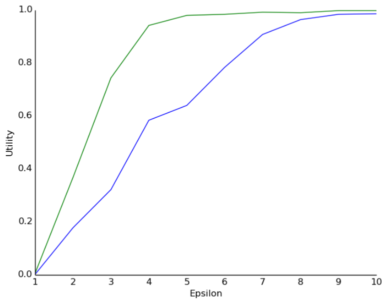

(b) $m_{ret} = 5$

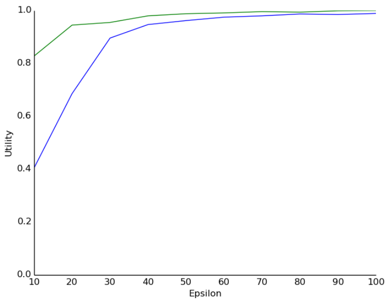

(c) $m_{ret} = 10$

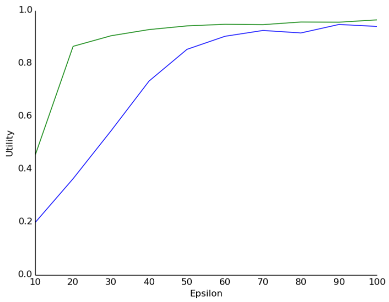

(d) $m_{ret} = 15$

Figure 4: We measure the performance of the Laplacian method for picking top SNPs in scenarios 1 (in blue) and 2 (in green) with m_{ret} (the number of SNPs being returned) equal to a. 3 b. 5 c. 10 and d. 15 for varying values of ϵ. For $m_{ret} = 3, 5$ we consider ϵ between 1 and 10, while in the other cases we consider ϵ between 10 and 100. We see that in all four graphs that scenario 2 leads to the best performance. Scenario 1, which is the one that appeared in previous work, leads to a greater loss of utility. These results are averaged over 100 iterations.

References

[Jian *et al.*, 2014] Jiang, X. *et al.* (2014) A community assessment of privacy preserving techniques for human genomes. *BMC Medical Informatics and Decision Making*, **14**, S1.

[Plenge *et al.*, 2007] Plenge, R. *et al.* (2007) TRAF1-C5 as a Risk Locus for Rheumatoid Arthritis– A Genomewide Study. *New England Journal of Medicine*, 1199-1209.

[Uhler *et al.*, 2013] Uhler, C. *et al.* (2013) Privacy-preserving data sharing for genome-wide association studies. *Journal of Privacy and Confidentiality*, **5**, 137-166.

[Yu and Ji, 2014] Yu, F. and Ji, Z. (2014) Scalable privacy-preserving data sharing methodology for genome-wide association studies: an application to iDASH healthcare privacy protection challenge. *BMC Medical Informatics and Decision Making*, **14**, S1.

[Yu *et al.*, 2014] Yu, F. *et al.* (2014) Scalable privacy-preserving data sharing methodology for genome-wide association studies. *JBI*, **50**, 133-141.
